# High-Performance pH Sensor Electrodes Based on a Hexagonal Pt Nanoparticle Array-Coated Nanoporous Alumina Membrane

**DOI:** 10.3390/ma15196515

**Published:** 2022-09-20

**Authors:** Abeer S. Altowyan, Mohamed Shaban, Asmaa Gamel, Ahmed Gamal, Mona Ali, Mohamed Rabia

**Affiliations:** 1 Department of Physics, College of Science, Princess Nourah bint Abdulrahman University, P.O. Box 84428, Riyadh 11671, Saudi Arabia; 2 Nanophotonics and Applications (NPA) Lab, Department of Physics, Faculty of Science, Beni-Suef University, Beni-Suef 62514, Egypt; 3 Physics Department, Faculty of Science, Islamic University of Madinah, P.O. Box 170, Madinah 42351, Saudi Arabia; 4 Nanomaterials Science Research Laboratory, Chemistry Department, Faculty of Science, Beni-Suef University, Beni-Suef 62514, Egypt

**Keywords:** pH sensor, porous anodic alumina membrane, Pt nanoparticles, Nernstian slope

## Abstract

Porous anodic alumina membranes coated with Pt nanoparticles (PAAM/Pt) have been employed as pH sensor electrodes for H^+^ ion detection. The PAAM was designed using a two-step anodization process. Pt nanoparticles were then sputtered onto the membrane at different deposition times. The membrane’s morphological, chemical, and optical characteristics were carefully assessed following the fabrication stage using a variety of analytical techniques. The potential of the PAAM/Pt sensor electrode was investigated by measuring the potential using a simple potentiometric method. The effects of depositing Pt nanoparticles for 3–7 min on sensor electrode sensitivity were examined. The optimal potentiometric Nernstian response slope for the PAAM/Pt sensor electrode with 5 min Pt sputter coating is 56.31 mV/decade in the pH range of 3.0 to 10 at 293 K. Additionally, the PAAM/Pt sensor electrode’s stability and selectivity in various ions solutions were examined. The sensor electrode had a lifetime of more than six weeks and was kept in a normal air environment.

## 1. Introduction

Researchers and scientists are interested in ordered porous structures because they have many fascinating characteristics [1,2,3]. The surface porosity, particularly the order surface, has a high surface that increases the interaction between the active sites and the electrolyte [4,5]. Fabricating nanostructures with good alignment and uniform size to actualize most of the proposed applications by the conventional lithographic process was even more difficult [6]. Various experiments have been conducted to create porous materials using naturally occurring materials or self-organizing materials such as carbonates [7,8,9,10,11]. Porous anodic alumina membranes (PAAMs), which are incredibly porous and have specified shapes and sizes, are one sort of ordered porous structure [12,13]. They may be modified to create free-standing membranes and have controlled pore density and periodicity [14]. These membranes are typical self-ordered nanoporous materials manufactured by Al with remarkable purity using a two-step anodization process. They are shaped in different forms with cylindrical holes in the centre. The PAAMs attracted much interest as a method for making nanowires, nanotubes, nanorings, and nanocones [1,2].

There have been many different types of biosensors discovered during recent decays, with and without nanoparticles, that operate according to a variety of hypotheses (changes in magnetic properties, refractive index, etc.) [15,16]. H^+^ ion detection was carried out using glass electrodes, metal oxide film, fluorescent pH, and fibre optical pH sensors [17,18]. Although these sensors offer numerous benefits, their use is constrained by a number of drawbacks [19,20]. pH determination is an important process for chemical, medical, and industrial applications. Only a few of the crucial aspects of life that depend on pH sensing include drinking water, food, biological contamination, and environmental contamination [21,22]. Some pH-sensing techniques, such as the glass electrode, are expensive and can only be used with very acidic and fluorine solutions. In addition, certain techniques—such as using metal oxides—are dangerous. Additionally, fluorescent pH sensors are only used for biological detections. These drawbacks encourage researchers to create new pH detecting techniques that are both highly efficient and affordable [23,24]. Recent research on various materials has been carried out to address the aforementioned problems. In prior studies, we used poly m-toluidine to effectively identify H^+^ ions in the pH range of 6–10 [25]. To determine pH, other investigations utilized a different type of polymer-coated with Au-Zn [26]. Additionally, the pH range of 7.6 to 8.2 was well-detected in ocean water by spot fluorescence. Moreover, limited studies have been focused on the use of nanoporous structures as sensitive elements for pH [27,28,29,30]. Ibupoto et al. developed NiO nanoporous structure on Au/glass substrate with a sensitivity of −43.74 ± 0.80 mV/pH and a quick response time of <10 s to test various phosphate buffered saline solutions in the pH range of 2–12 [27]. According to McMurray et al., a porous RuO_2_-glass composite has been used as a pH sensor and exhibits near-Nernstian potential with a response time of ~90 s and a maximum hysteresis of 30 mV in the pH range of 2–12 [28]. In 2020, Manjakkal et al. highlighted the developments and prospects for electrochemical pH sensors based on metal oxide nanostructures [29]. Recently, plasmonic nanostructures have been applied to improve pH-sensing parameters [31,32]. Chu et al. used Pt nanoparticles to enhance the pH-sensing characteristics of ZnO nanorods [32]. Therefore, it is crucial to design a pH-sensitive element based on arrayed nanoporous structures, such as PAAM coated with plasmonic nanostructure, and to study how it responds to various pH levels.

In this study, a two-step anodization procedure and the sputter coating technique were used to create Pt/PAAM sensors of different Pt sputtering times, and they were then characterized by using various analytical instruments. The influence of the Pt coating time on the sensitivity and selectivity of the PAAM/Pt sensor to detect the proton ion was evaluated. Additionally, the PAAM/Pt sensor’s lifetime and stability were investigated.

## 2. Experimental Details

### 2.1. Materials

From Sigma Aldrich in Germany, high purity Al foils (99.999%), high purity sulfuric, phosphoric, and oxalic acids were purchased. From Biochem in Egypt, chromium oxide CrO_3_ (99.9%) was acquired. The m-toluidine, NaOH, KCl, HCl, and citric acid (C_6_H_8_O_7_) were purchased from ElNaser co., Egypt. Na_2_B_2_O_7_ and Na_2_HPO_4_ were obtained from United co. Egypt. Buffer solutions were prepared in our lab using a certain percentage of the reagent, in which buffer solutions of pH 1 and pH 2 were prepared from KCl and HCl, and buffer solutions of pH 3 to pH 8 were prepared from Na_2_HPO_4_ and C_8_H_8_O_7_. Moreover, buffer solutions of pH 9 and pH 10 were prepared from Na_2_B_2_O_7._

### 2.2. Fabrication and Characterization of the PAAM/Pt Sensor Electrode

Nanoporous Anodic Alumina Membranes (PAAMs) were made using a two-step anodization technique [2]. High purity Al foils (99.999%, 0.25 mm thickness, Merck, UK) were employed as the starting material. These Al foils were cleaned for 10 min in deionized water at 25 °C after being degreased in 99.9% acetone for 5 min and 99.9% methanol for 5 min at 150 °C. The foil was electropolished for three minutes at 50 °C with steady stirring in a solution of H_3_PO_4_:H_2_SO_4_:H_2_O (1:1:1). The clean Al foil was first anodized in a 0.3 M C_2_H_2_O_4_ solution at 50 V and 7 °C for 13 h. After first being anodized, the alumina membrane was submerged for three hours at room temperature in a solution of H_2_O, H_3_PO_4_, and CrO_3_ (100 mL:10 mL:1 g). The second anodization took place afterwards for 5 min under the same conditions as the first anodization. The created PAAM was submerged in a pore-widening solution (6%wt phosphoric solution) for 45 min at room temperature (25 °C).

At a pressure of 2 torr, a current of 6 mA, and a separation of 8 cm from the Pt target, Pt was deposited on the top surface of PAAM using a fairly simple sputter-coating process. The deposition time of Pt varied from 0 to 7 min.

Using an energy-dispersive X-ray spectrometer (EDX) and a field emission scanning electron microscope (FE-SEM; models: ZEISS SUPRA 55 VP and ZEISS LEO, Gemini Column), the morphologies and constituents of the synthesized PAAM and PAAM/Pt samples were identified. The gathered samples’ reflection spectra were measured from 250 to 2500 nm using a UV-Vis-NIR spectrophotometer (model: LAMBDA 950, Perkin Elmer, Waltham, MA, USA). A two-electrode cell was used for sensing measurements, with the synthesized PAAM/Pt acting as the working electrode in the presence of the saturated calomel electrode as a reference electrode. The measurements were made at 25 °C using a multimeter (UT71A, Uni-Trend Technology, Dongguan, China). By using a number of buffer solutions with pH ranging from 1 to 12, the effects of depositing Pt nanoparticles for 3 to 7 min on sensor electrode sensitivity were evaluated. In addition, the lifetime and response time were examined.

## 3. Results and Discussion

### 3.1. Characterization of PAAM and PAAM/Pt

**Figure 1:**
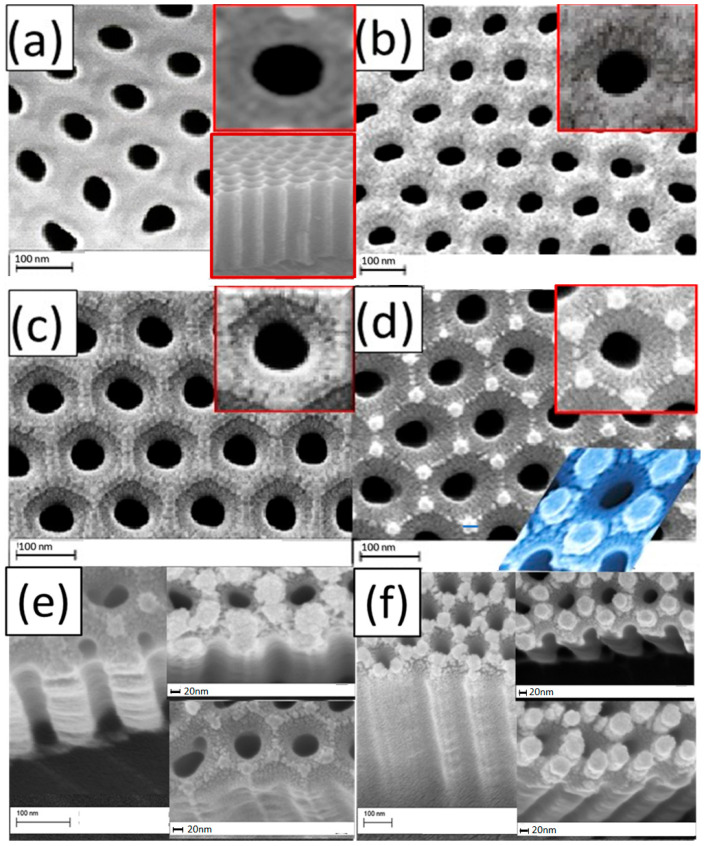
FE-SEM images of PAAMs pore widened for 45 min and sputter coated at current 6 mA for different deposition times; (**a**) 0, (**b**) 3, (**c**) 5, and (**d**) 7 min; (**e**,**f**) cross-section and oblique view images of Pt/PAAMs membranes. Insets of (**a**–**d**) showed enlarged images of a single cell, in addition to the cross-section image of PAAM without coating in (**a**).

To investigate the effect of Pt sputtering time on the morphology of the PAAM, a series of identical PAAMs (pore-widened of 45 min) was coated with Pt nanoparticles for 0, 3, 5, and 7 min (Figure 1a–d), and this coating process carried out using sputter device at low-vacuum conditions. The PAAM after widening has a pore diameter of 56 nm and an inter-pore distance of 125 nm. The PAAM has a hexagonal shape with high-pore ordering with a pore density of 1.01 × 10^10^ cm^−2^ and porosity of 18%. A cross-section image of PAAM without coating is shown in the inset of Figure 1a. In addition, the PAAM has six active regions (effective regions) around each pore, and this provides a high surface area, as we illustrated in our previous study [33].

The surface of PAAM is fully coated with Pt, as shown in the FE-SEM images of Figure 1b. The pore diameter after 3 min coating is reduced to 53 ± 3 nm. After 5 min of coating (Figure 1c), the thickness of the Pt layer increases and selectively grows over the inner wall at the hexagonal boundaries between the PAAM cells, which consist of high-purity alumina. The pore diameter after 5 min coating is reduced to 51 ± 2 nm. The Pt growth increased around the active sites, which serve as seeds for this growth, with the appearance of sub-gaps when the deposition time increased to 7 min (Figure 1d). The top and oblique blue insets of Figure 1d show the hexagonal array of Pt nanodiscs around each nanopore. The pore diameter after 7 min coating is reduced to 49 ± 1.0 nm, and the diameter of the Pt discs is 27.5 ± 1.5 nm. The presented morphologies are expected to be very important for sensing applications because they can support the chemical and electromagnetic enhancement of the detected signals. Until now, many reports have been published for the sensing applications of Au/PAAM and Ag/PAAM nanostructures [34], but the pH sensing application of the presented morphologies in Figure 1b–d has not been reported to the best of our knowledge. The FE-SEM images in Figure 1e,f show cross-sectional and oblique view images of the coated membranes to confirm the entrance of Pt in PAAM’s pores, as the porous support was used to increase the surface area and improve sensing parameters.

**Figure 2:**
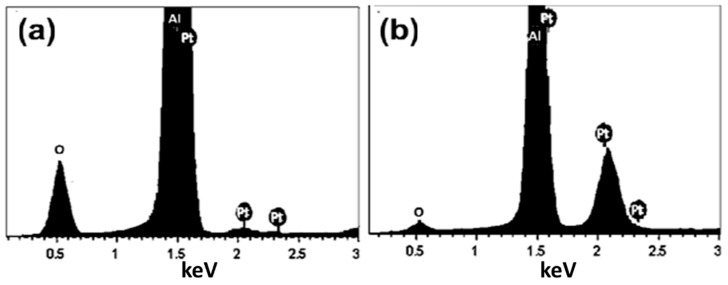
EDX spectra of Pt/PAAM samples coated with Pt for (**a**) 3 min and (**b**) 7min.

Pt depositions on the PAAM against sputtering time were qualitatively evaluated using an energy dispersive X-ray spectrometer (EDX). Figure 2a,b show the EDX spectra of Pt/PAAM samples coated with Pt for 3 and 7 min. This figure displays the Al, O, and Pt signals. The quantitative analysis showed that the Pt mass% significantly increased from 3.1% to 16.1% when the sputtering period changed from 3 to 7 min. Our EDX results confirmed that the deposited Pt nanostructures had excellent purity because of the high degree of un-reactivity of Pt.

**Figure 3:**
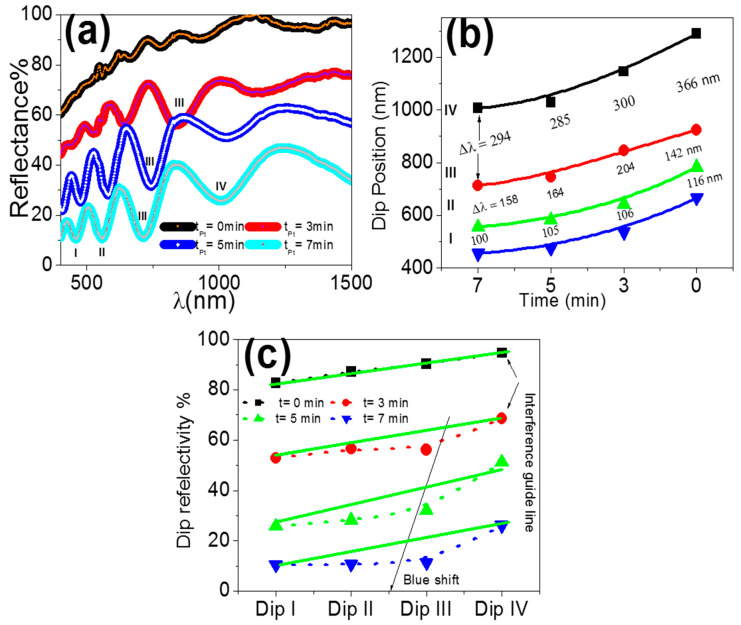
(**a**) Reflection spectra of PAAMs/Pt at different deposition times, (**b**) the variation of dip position, and (**c**) dip reflectance % as a function of the deposition time for dips I, II, III, and IV.

Optical reflectance analyses for the PAAM and PAAM/Pt (at different Pt coating times) are revealed in Figure 3a. The reflectance spectra are about 70–100% after 400 nm, in which the scattering and scattering coefficient are very small after 400 nm. The observed high peaks and dips caused by the reflectance spectra’s constructive and destructive interferences with the reflected waves constitute evidence of the PAAM’s well-built structure. When wavelengths increased, the contrast was lower and the peak’s width was larger. The PAAM/Pt reflectance spectra exhibit stronger and sharper fringes that shift to shorter wavelengths, just as the PAAM spectra. When Pt deposition duration is prolonged from 0 to 7 min, peak interference contrasts increase and reflectivity decreases. Figure 3b shows the exponential connection between the Pt deposition time and dip position, with an increasing blue shift as the Pt deposition time increases. In addition, the wavelengths and the separation between dips, Δλ, become smaller as deposition time increases. Figure 3c illustrates the link between deposition time and dip reflectance, with dips I and IV exhibiting a linear drop and dips II and III exhibiting a nonlinear behaviour. The Maxwell–Garnet medium approximation shows that dielectric constants and pore width vary with deposition time [2,35]. At 3 min deposition time, interference dip III is noticeably stronger than the others. In combination with the interference fringe at 846 nm, this may indicate the existence of a localized Pt surface plasmon [36,37]. By increasing the deposition time, the surface plasmon is blue-shifted, as indicated in Figure 3c.

### 3.2. Sensing Properties

Different buffer solutions with pH ranges between 3.0 and 10 at 25 °C were used to study the calibration curves of the PAAM coated with various Pt nanoparticle thicknesses as a function of coating time. In addition, four distinct solutions of pH 7 are used to study the sensor’s selectivity for H^+^ ions, and each solution’s relative standard deviation is calculated. Finally, the sensor electrode lifetime is investigated by utilizing the same calibration measurement behaviour over a buffer of pH 3.0–10 for several weeks. The calibration curves for the sensor are based on the Nernst Equations (1) and (2), where *n* represents oxidation electrons, *Q* represents H^+^ ions concentrations, *T* represents temperature in kelvin, *R* represents a universal gas constant, and *F* represents the Faraday constant, with *RT*/*F* = 0.0591 at 25 °C [36,37,38,39].

(1) $$E=Eo-\frac{RT}{nF}\ln Q$$

(2) E=Eo+0.0591 pH

#### 3.2.1. Sensitivity of PAAM Sensor to H^+^ Ions under the Effect of Different Pt Film Thicknesses

**Figure 4:**
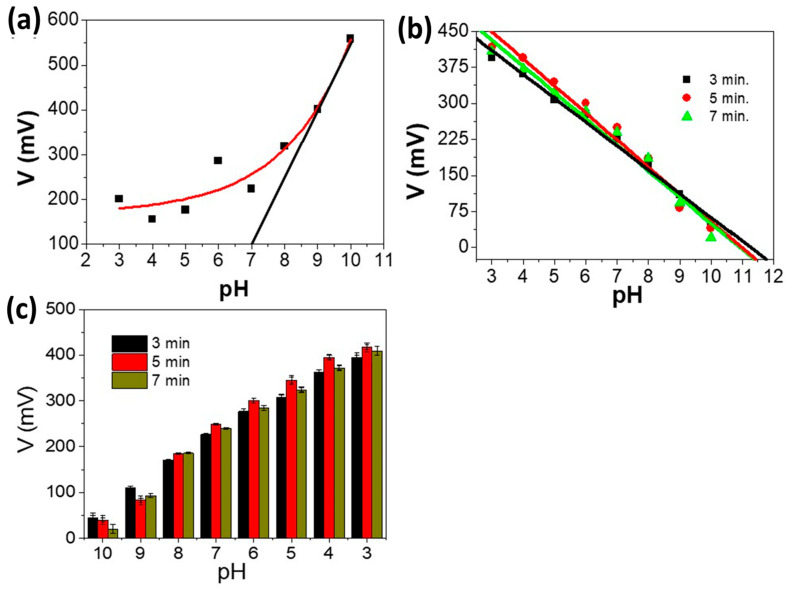
Calibration curve of (**a**) PAAM, (**b**) PAAM/Pt sensors electrode, and (**c**) average hysteresis with different Pt coating times of 3, 5, and 7 min at 293 K.

The sensitivity (potentiometric response slopes) of PAAM and PAAM/Pt to H^+^ ions is studied using the simple potentiometric method based on Equation (2) [10,11,40,41]. Table 1 summarizes the information collected from Figure 4a,b, which display the potentiometric response slopes (buffer 3 to 10) for PAAM and PAAM/Pt sensor electrodes with various Pt thicknesses under study. The relationship between pH and the potentiometric slope for the PAAM is an exponential curve. Therefore, the potentiometric response slope of the PMMA as a pH sensor cannot be determined using Nernstian behaviour. This implies that the PAAM is useless as a standalone pH sensor. At pH values ≥ 7, it seems that the Al/PAAM/liquid can act as a semiconductor junction, and the band-gap barrier potential can be overcome. The noble Pt plates have been used to improve the sensitivity of PAAM to H^+^ ions.

The potentiometric response slope values for the PAAM/Pt sensors vary depending on the Pt film thicknesses as a function of the Pt sputter-coating duration, which ranges from 3 to 7 min. The potentiometric response slope increases from 49.56 to 56.31 mV/decade when the Pt sputter-coating time extended to 5 min, but when the Pt time coating increased to 7 min, the slope decreases to 54.47 mV/decade. This shows that the generated E value for the sensor with a 5 min Pt coating time and the change in H^+^ concentrations are more consistent with the Newtonian theory (Equation (2)). From this sensor, the experimental slope of 56.31 mV/decade is closer to the theoretical value of 59.1 mV/decade (Equation (2)). However, Pt coating with times at 3 or 7 min is less close to the theoretical Nernstian value.

The change in the potentiometric response slope is ascribed to the change in the sensitivity of the PAAM/Pt sensor electrode to proton ions that depends on Pt film thicknesses [42,43]. From Figure 4 and Table 1, the optimum potentiometric response slope is for the sensor with a Pt film of 5 min sputtering time. This PAAM/Pt sensor electrode can function with good behaviour in weak acid or base conditions with a good pH range (3–10). In addition, the detection limit (DL) is determined for the PAAM/Pt sensor under different Pt coating, in which the DL values are 11.77, 11.47, and 11.42 for Pt coatings of 3, 5, and 7 min, respectively. These values are calculated by extrapolating the data line until the intercept with the X-axis [44,45]. The average calibration curve points (hysteresis) are represented in Figure 4c. The pH 7 and pH 8 have potential values that are closer together. This suggests that these pH levels are ideal for the sensor. At pH 7 and 8, the standard deviation values are typically around 1.5%.

#### 3.2.2. PAAM/Pt Sensor Electrode Selectivity

The selectivity of the PAAM/Pt sensor electrode to proton ions is studied in different ions solutions (H^+^, Na^+^, K^+^, and Mg^2+^) of the same concentrations at 293 K [46,47,48,49,50]. From the different solutions, the sensor potential is calculated for each one and then compared with the main H^+^ ion value (distilled H_2_O) of pH 7. The results in Table 2 show an average recovery of 93.8% with a total Relative Standard Deviation (RSD) of 4.3%. Moreover, with the presence of other interfering ions, these results confirm the utility of the proposed PAAM/Pt sensor electrode for H^+^ ion detection.

#### 3.2.3. PAAM/Pt Sensor Electrode Stability

**Figure 5:**
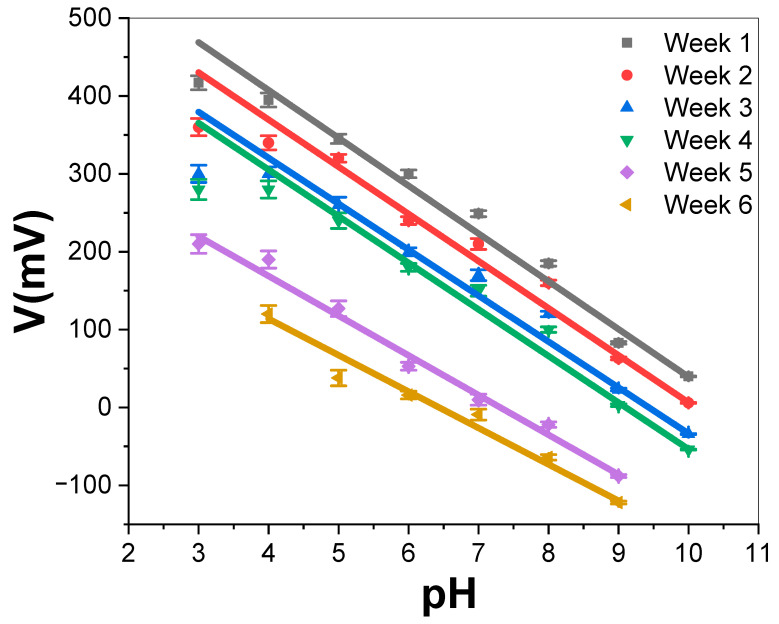
Calibration curves of PAAM/Pt sensor electrode for different weeks at 293 K.

Every three days, the PAAM/Pt sensor’s potentiometric response slope is tested in buffer solutions with a pH range of 3.0 to 10, as shown in Figure 5. During measurements, the prepared sensor is reintroduced into the buffer solution after being kept in ambient air. The sensor electrode is thoroughly dried at a temperature of around 40 °C after measurement processes and then cleaned with deionized water for storage. All the data obtained from Figure 5 are listed in Table 3. The sensor’s potentiometric response slope over the six weeks matches the Nernstain slope with a little change from 56.31 to 49.47 mV/decade between the first and sixth weeks. Moreover, the sensor electrode has potentiometric response slopes of 51.2 and 44.02 mV/decade in the first and sixth weeks, respectively, with buffer solutions of pH changes from 3 to 10. In addition, the sensor response range decreases from 3–9 in the fifth week to 4–9 in the sixth week. After that, there is more change in the potentiometric slope of the Nernstian value due to some Pt etching from PAAM’s surface [51,52].

#### 3.2.4. Response Time and Reproducibility

**Figure 6:**
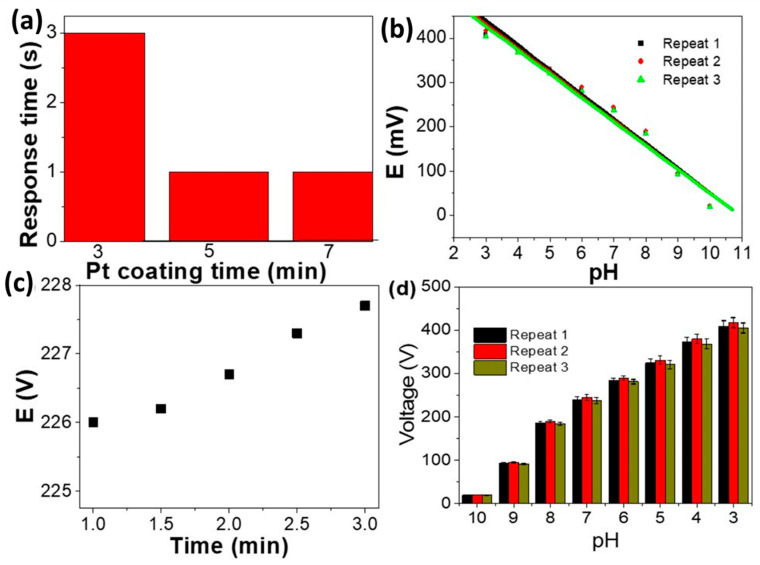
(**a**) Response time for PAAM/Pt sensor at different Pt coating times from 3 to 7 min, (**b**) reproducible studies, (**c**) time effect, and (**d**) reproducible studies and error bars for the PAAM/5 min-Pt sensor.

The response time is the time a sensor needs to reach 90% of its final potential value, in which there is contact between the sensor and ions [53,54,55,56]. Figure 6a shows the relationship between the PAAM/Pt response time and the Pt coating time (3–7 min). For PAAM coated with Pt, the response times are 3, 1, and 1 s for deposition durations of 3, 5, and 7 min, respectively. As the Pt coating becomes thicker, the reaction time slows down. This behaviour highlights how crucial a Pt coating is for enhancing a sensor electrode’s sensitivity and for enabling accurate H^+^ ion detection. In addition to this, a PAAM/Pt (5 min coating) reproducibility study was carried out and mentioned in Figure 6b for buffer solutions of pH 3 to pH 10.

The calculated different pH parameters are calculated and mentioned in Table 4 for the sensor. According to Figure 6 and Table 4, the potentiometric response slope with cycles (repeating) shows little variations, with values changing from 56.31 to 55 mV/decade from cycle 1 to cycle 3, respectively. Additionally, Figure 6c mentions the sensor electrode’s performance for 3 min. With a standard deviation of 1.01% and a relative standard deviation of 0.45%, the sensor has high operating time stability for three minutes. Additionally, Figure 6d mentions the reproducible experiments and error bars to support the excellent reproducibility studies for the sensor. Finally, a comparison study of the pH-sensing parameters for the PAAM/Pt sensor electrode with previously reported values for other materials is mentioned in Table 5. From this table, the newly prepared sensor has more sensing properties than our previous sensor based on Au/poly(m-toluidine), in which the new prepared sensor works in a pH response range of 3–10 with a Nernstian response slope of 56.31 mV/decade [25]. Moreover, from the table, other sensing studies have many drawbacks in terms of pH response ranges or Nernstian behaviour [19,20,57,58,59,60]. As a result, the newly produced sensor may be used in industrial H^+^ detection processes.

## 4. Conclusions

A PAAM/Pt sensor electrode was constructed using the fundamental potentiometric method to find H^+^ ions in aqueous solutions. The sensor was made using a two-step anodization process and the PAAM surface was coated with Pt nanoparticles using a sputtering technique. Six active dots served as a seed for Pt growth on the PAAM surface surrounding the PAAM pore in the optical and SEM experiments that showed the effects of Pt on the enhancement of the reflectance and active sites for PAAM. The average reflectivity decreases and the peak contrast increases with longer Pt deposition durations and a blue shift in reflective dip areas because the formed Pt nanostructures coincide with surface plasmon resonance. By using the detection process, many parameters were studied: the coating time of the Pt on PAAM, and the selectivity and stability of the PAAM/Pt sensor. The optimal coating Pt time was 5 min, with a Nernstian slope in the buffer range of 3 to 10 of 56.31 mV/decade. Additionally, the selectivity under examination had an RSD of 4.3%. The sensor’s lifetime was around six weeks. This study merely highlights the lab-scale usability of the suggested sensor. Future work will focus on re-imaging the sensor to determine whether or not the Pt or PAAM substrate was affected by low pH solutions. Several sensor-restoration approaches and sensor topologies will be described to enhance the Nernstian slope and durability of the sensor for industrial real applications. Moreover, our future study will seek to focus on the real-world applications of the optimized sensor in food processing, health monitoring, agricultural, and water quality monitoring.

## Figures and Tables

**Table 1:** Summarized data for PAAM/Pt sensors at different Pt film coating times from 3 to 7 min in pH solutions (pH 3.0–10) at 293 K.

Time Coating (min.)	-Slope (mV/Decade)	R^2^	Response Range	Detection Limit
3	49.65	0.989	3–10	11.77
5	56.31	0.974	3–10	11.47
7	54.47	0.974	3–10	11.42

**Table 2:** PAAM/Pt sensor selectivity to proton ions in different ion solutions.

Ions	V (mV)	RSD %
H^+^	250	0
Na^+^	233	4.91
K^+^	230	4.97
Mg^2+^	225	7.44

**Table 3:** The summarized data for PAAM/Pt sensor electrode as a function of the number of weeks at 293 K.

Lifetime (Week)	-Slope (mV/Decade)	R^2^	Response Range
1	56.31	0.987	3–10
2	52.1	0.980	3–10
3	49.7	0.997	3–10
4	49.67	0.977	3–10
5	51.2	0.990	3–9
6	44.02	0.98	4–9

**Table 4:** The calculated different pH sensing parameters for PAAM/5 min-Pt from Figure 6b.

Repeating Times	-Slope (mV/Decade)	R^2^	Response Range
1	56.31	0.989	3–10
2	55.56	0.98	3–10
3	55.0	0.987	3–10

**Table 5:** A comparison study of the pH sensing parameters for the PAAM/Pt sensor with previously studied materials.

pH Sensor Electrode	Slope mV/Decade	Response Range	Ref
Au/poly(m-toluidine)	57.34	6–10	[25]
ZnO/Si	22.4	4–11	[60]
Chitosan	56.0	2–9	[59]
CuS	27.8	2–12	[58]
SiO_2_	53.0	1–9	[57]
ZnO/Pd/ZnO	52.0	5–12	[20]
ITO membrane	36.4	4–10	[19]
PAAM/5 min Pt	56.31	3–10	This work

## Data Availability

Not applicable.
